# *In silico* physicochemical characterization and topology analysis of Respiratory burst oxidase homolog (Rboh) proteins from Arabidopsis and rice

**DOI:** 10.6026/97320630014093

**Published:** 2018-03-31

**Authors:** Gurpreet Kaur, Pratap Kumar Pati

**Affiliations:** 1 Department of Biotechnology, Guru Nanak Dev University (GNDU), Amritsar 143005, Punjab, India; 2 Max Planck Institute for Developmental Biology, Tuebingen 72076, Germany

**Keywords:** plant NADPH oxidase, *in silico*, physicochemical characterization, subcellular localization, signal peptide, topology

## Abstract

NADPH oxidase (NOX) is a key enzyme involved in the production of apoplastic superoxide (O2-), a type of reactive oxygen species (ROS). Plant Noxes are the homologs of mammalian NADPH oxidase's catalytic subunit and are documented as respiratory burst oxidase homologs (Rbohs). A number of studies have reported their diverse functions in combating various stresses and in plant growth and development. In the present study, a total of 19 Rboh proteins (10 from Arabidopsis thaliana and 9 from Oryza sativa Japonica) were analyzed. We employed in silico approaches to compute the physiochemical properties (molecular weight, isoelectric point, total number of negatively and positively charged residues, extinction coefficient, half-life, instability and aliphatic index, grand average of hydropathicity, amino acid percentage). We observed a lot of variability in these parameters among the Rbohs accounting for their functional diversification. Their topological analysis, subcellular localization and signal peptide detection are also performed. To the best of our knowledge, the present study report on in silico physiochemical characterization, topology analysis, subcellular localization and signal peptide detection of Rboh proteins within two model plants. The study elucidates the variations in the key properties among Rbohs proteins, which may be responsible for their functional multiplicity.

## Background

The accelerated generation of reactive oxygen species (ROS) such as superoxide (O2-), singlet oxygen (1O2), and hydrogen peroxide (H2O2) has been implicated as one of the earliest hallmark of plants stress response. The major source of ROS production in plants is NADPH oxidase, which is localized to the plasma membrane and transfer electrons from cytosolic NADPH/NADH to apoplastic oxygen leading to ROS. It is the homolog of the mammalian NADPH oxidase catalytic subunit known as gp91phox [1]. In contrast to animals, plant NADPH oxidase consists of two main structural elements: Respiratory burst oxidase homologue (Rboh) and Rop (Rho-like protein; a Rac homologue of plants). OsRbohA was the first plant NADPH oxidase identified in Oryza sativa [2] and now plant NADPH oxidases encompass several Rbohs in dicots, monocots and lower plants [1]. Rboh proteins consist of two Ca 2+-binding EF-hand motifs in the N-terminal region, six transmembrane helices and FAD and NADPH binding domains in the C-terminal. Recently available crystal structure of OsRbohB N-terminal region (138- 313 amino acid residues) has highlighted the presence of two additional EF-hand-like motifs (EF-like 1 and EF-like 2) [3]. Rbohs perform ambidextrous functions in plant growth, development, and responses to abiotic and biotic stresses. The functioning of Rbohs requires interaction with various regulatory components which involve Ca2+, calcium-dependent protein kinases (CDPKs), Ca2+/CaM-dependent protein kinase (CCaMK), Rop, extracellular ATP (eATP), phospholipase Dα1 (PLD α1) and its lipid product phosphatidic acid (PA), mitogen activated protein kinase (MAPK), Nt14-3-3h/omega1 (a member of 14-3-3 protein family) and nitric oxide [1] . As evident from various studies, ROS production by Rbohs is associated with numerous stress, morphogenesis and development bound signalling pathways; although, how this ROS wave is deciphered downstream for a particular response is still to be elucidated. The study of various physiochemical parameters may provide the insight into their functional diversity.

Besides an array of experimental techniques available, various in silico approaches and online tools provide enormous opportunities for the characterization and analysis of gene and protein sequences [4, 5]. These tools provide researchers a cost effective and faster output to understand genes and proteins, which will help in designing lab experiments. Recently, we have conducted phylogenetic analysis of Rbohs within the plant kingdom with orthologous identification, mutation and disorder prediction [6]. Further, an in silico study for the analysis of ciselements, CpG islands and tandem repeats on upstream regions from Rbohs of Arabidopsis thaliana and Oryza sativa japonica to get insights into their versatile functions was also carried out [7]. In addition to this, some non-homology based approaches such as physio-chemical parameters, subcellular localization, signal peptide prediction etc., may also provide useful insights into the functional diversity of proteins [5]. Several physicochemical properties of a protein such as isoelectric point, molecular weight, number of negatively and positively charge amino acid residues, instability index, aliphatic index and grand average of hydropathicity (GRAVY) can be computed. Various experimental studies have indicated the expression of Rbohs in plants including few from Arabidopsis thaliana and Oryza sativa. 10 Rbohs from A. thaliana and 9 from O. sativa have been reported, but the information regarding their biological role to various abiotic (cold, drought, osmotic, salt, heat and light) and biotic (pathogens and herbivores) stresses is still incomplete [1]. To the best of our knowledge, no study has been documented yet on the physiochemical characterization and topology analysis of Rbohs.

## Methodology

### Sequence retrieval

Accession numbers of protein sequences for Arabidopsis and rice Rbohs were retrieved from a recent study of our lab [1]. A total of 19 sequences (10 from Arabidopsis and 9 for rice) were downloaded from UniProt (http://www.uniprot.org/) in FASTA format and used for further analysis.

### Physio-chemical properties

The physicochemical properties were computed for 19 Rboh proteins using the ExPASy ProtParam tool (http://web.expasy.org/protparam/) [8]. Web servers specialized in predicting cellular localization of protein sequence were used: WoLF PSORT (http://wolfpsort.seq.cbrc.jp/) [9], CELLO v.2.5 (http://cello.life.nctu.edu.tw/) [10] and EuLoc (http://euloc.mbc.nctu.edu.tw/) [11]. For signal peptide detection, SignalP 4.1 Server (http://www.cbs.dtu.dk/services/SignalP/) [12] and PrediSi (http://www.predisi.de/) [13] were employed.

### Topological analysis

Topological analysis of individual Rboh proteins were done using TMHMM (http://www.cbs.dtu.dk/services/TMHMM/) [14], Phobius (http://phobius.sbc.su.se/) [15], HMMTOP (http://www.enzim.hu/hmmtop/) [16] and WHAT (http://saier-144-21.ucsd.edu/barwhat.html) [17] programs. Sequences were aligned with ClustalX 2.0.11 ( http://www.clustal.org/clustal2/) [18] and their average hydropathy, amphipathicity and similarity were estimated using AveHas program (http://saier-144-21.ucsd.edu/baravehas.html) [19].

## Results

In the present study, various physio-chemical properties, subcellular localization, signal peptide detection and topological analysis of 19 Rboh protein sequences, 10 from Arabidopsis and 9 from rice were analyzed. The protein name and accession number are shown in Table 1.

### Physio-chemical properties

**Figure 1:**
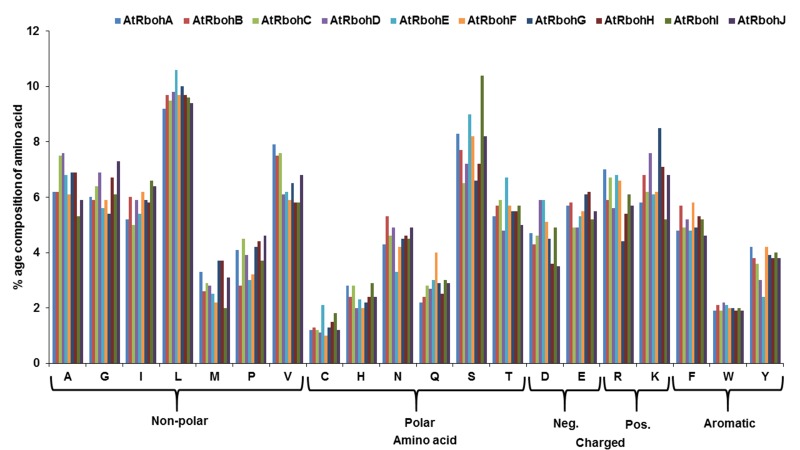
Amino acid percentage composition showing distribution for different types of amino acids in 10 AtRboh proteins computed using ExPASy ProtParam tool.

Physio-chemical properties were calculated for 19 Rboh proteins (Table 2). The properties include length, molecular weight, isoelectric point (pI), total number of negatively and positively charged residues, extinction coefficient, instability index (II), aliphatic index (AI) and grand average of hydropathicity (GRAVY). Among Arabidopsis Rbohs, AtRbohB was the shortest Rboh with 843 amino acids while AtRbohE is the longest one with 952 amino acids. The computed pI was more than 7 for all 10 AtRbohs, where the lowest (8.71) and highest (9.48) values were obtained for AtRbohI and AtRbohJ, respectively. The number of positively charged amino acids was more than negatively charged among all AtRbohs. Extinction coefficients (ECs) were determined at 280 nm with the assumption that all pairs of Cys residues form cystines. They were falling in the range of 143295 to 164600 M-1 cm-1 where the lowest value corresponds to two Rbohs; AtRbohC and AtRbohG, while highest value corresponds to AtRbohF. The instability index (II) for AtRbohs range from 38.32 (AtRbohD) to 48.99 (AtRbohI). In addition to II, aliphatic index (AI) for AtRbohs were also computed, whichwas found to vary from 83.88 (AtRbohH) to 89.37 (AtRbohB). The GRAVY score was observed in range from -0.16 (AtRbohB) to -0.241 (AtRbohD). Further, the amino acid percentage composition of 20 amino acids among 10 AtRbohs was determined (Table 3) and their distribution for different types of amino acids was determined (Figure 1).

**Figure 2:**
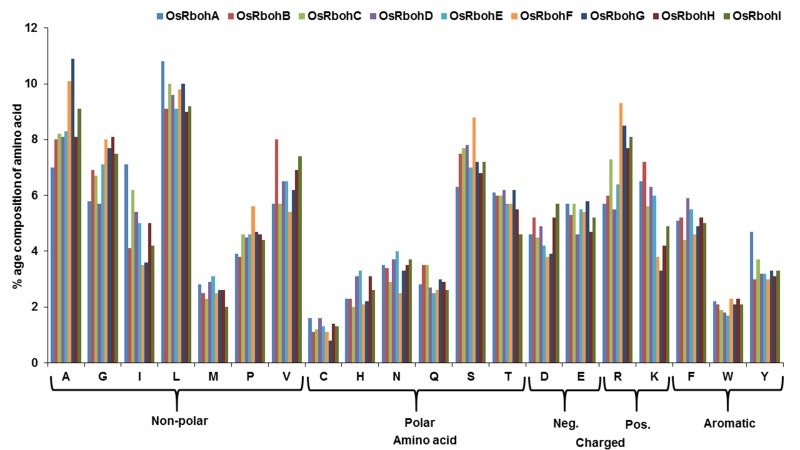
Amino acid percentage composition showing distribution for different types of amino acids in 9 OsRboh proteins computed using ExPASy ProtParam tool.

In case of rice Rbohs, OsRbohA was the shortest protein with 743 amino acids while OsRbohF was the longest one with 1033 amino acids (Table 2). The computed pI was >7 for all 9 OsRbohs where the lowest (8.98) and highest (9.84) values were obtained for OsRbohA and OsRbohF, respectively. The number of positively charged amino acids was more in number than negatively charged among all OsRbohs. Extinction coefficients were obtained in the range of 117855 to 165170 M-1 cm-1 where the lowest value corresponds to OsRbohE, while highest value corresponds to OsRbohG. The instability index (II) for OsRbohs range from 39.76 to 49.34. The highest II was observed for OsRbohF (52.79), which was followed by OsRbohC (50.23) and OsRbohG (49.34). However, the lowest II value was obtained for OsRbohB. The AI for OsRbohs was found to vary from 77.51 (OsRbohF) to 93.2 (OsRbohA). The GRAVY score lies in the range from -0.087 (OsRbohA) to -0.286 (OsRbohF). Further, the amino acid percentage composition among 9 OsRbohs (Table 3) and and their distribution for different types of amino acids were determined (Figure 2).

**Figure 3:**
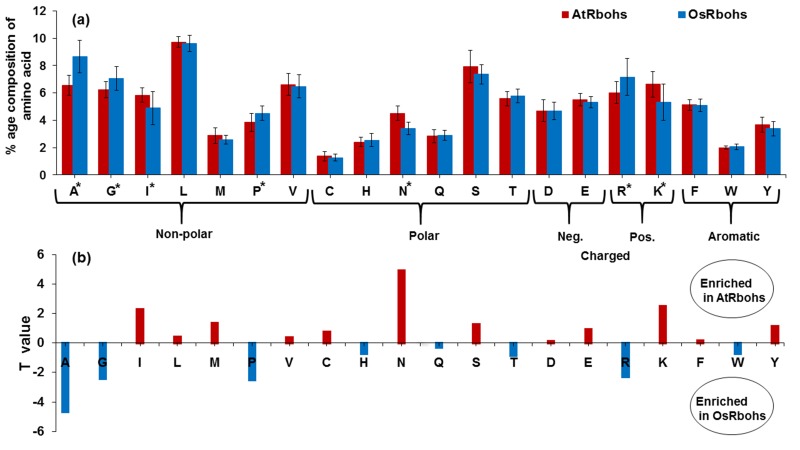
T-tests reveal significant differences between AtRboh and OsRboh proteins. (a) Average amino acids percentage composition found in AtRbohs (red) and OsRbohs (blue). Asterisks represent a statistically significant difference between the two averages; error bars are ± σ. (b) T-values from t-tests for the amino acids composition with significant difference between the two plants. The magnitude of the bar indicates the relative difference between the two means and the direction of the bar (up or down) indicates which plant contains the higher percentage of that amino acid.

To find any significant differences among amino acid composition between two species, t-tests were applied. They revealed significant differences between AtRbohs and OsRbohs in the percentage of non-polar (alanine: A, glycine: G, isoleucine: I and proline: P), polar (asparagine: N) and positively charged (arginine: R, lysine: K) amino acids (Figure 3a). The magnitude as well as direction of the significant differences in the amino acid percentage composition for the two species is represented by their t-test values in Figure 3b. The height of the bar indicates the relative difference in the sample means and its direction (up or down) represents which plant species contain the higher percentage of that amino acid. Positive t-test values indicate a higher percentage of that amino acid in AtRbohs whereas negative values correspond to a higher percentage in OsRbohs.

The estimated half-life for 18 Rbohs except OsRbohA was found to be 30 hours (mammalian reticulocytes, in vitro), >20 hours (yeast, in vivo) and >10 hours (Escherichia coli, in vivo). For OsRbohA, it was 4.4 hours (mammalian reticulocytes, in vitro), >20 hours (yeast, in vivo) and >10 hours (E. coli, in vivo). Subcellular localization prediction indicated all 19 Rbohs as plasma membrane associated and absence of any signal peptide.

### Topological analysis

**Figure 4:**
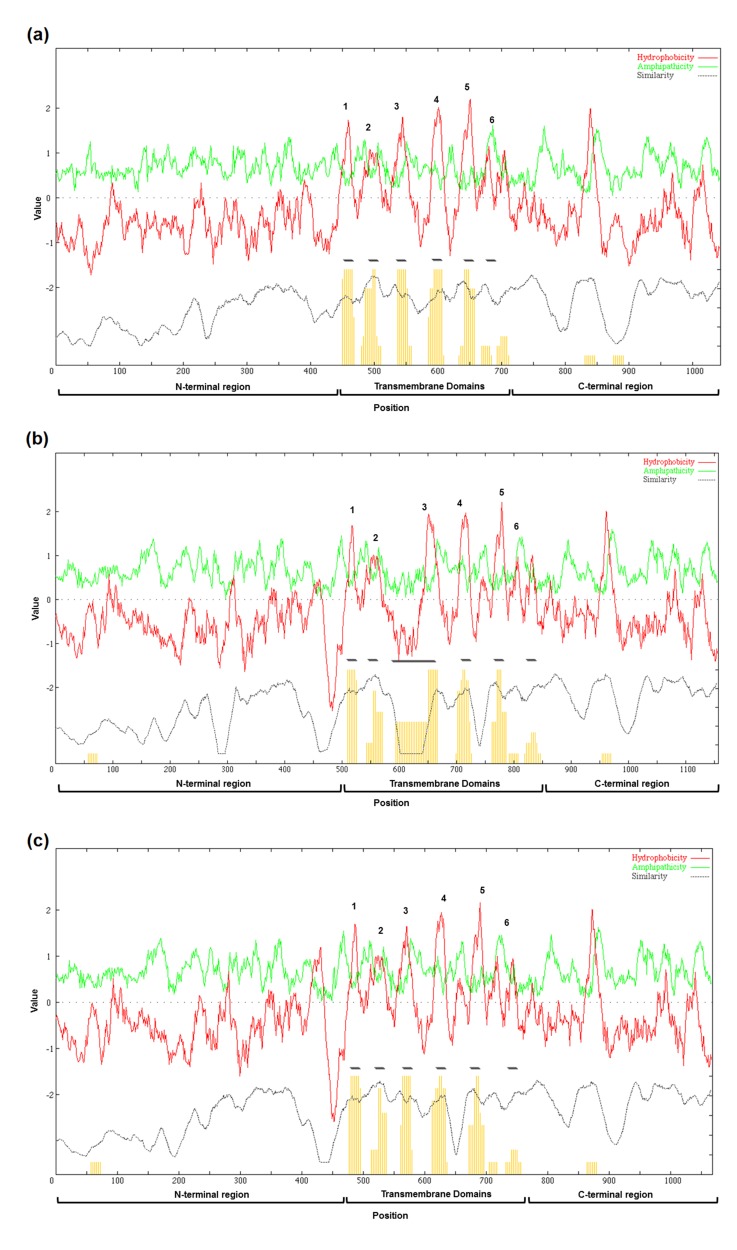
The average hydropathy, amphipathicity and similarity plots generated for (a) 10 AtRbohs and (b) 9 OsRbohs and (c) 8 OsRbohs using AveHAS program. Top red and green lines indicate average hydropathy and average amphipathicity, respectively. Bottom dotted lines denote average similarity. Six TMDs are shown with yellow bars. Hydrophobicity peaks for six TMDs are indicated in numbers.

Individual Arabidopsis and rice Rboh proteins were predicted to contain 4 to 7 transmembrane domains (TMDs) based on TMHMM, Phobius, HMMTOP and WHAT programs. However, more accurate results could be obtained when aligned homologous sequences are used. Hence, multiple sequence alignments were done for 10 AtRboh and 9 OsRboh proteins (S1 File) to generate average hydropathy, amphipathicity and similarity plots (Figure 4a & b). Hydropathy refers to the extent of hydrophobicity or hydrophilicity of amino acids while amphipathicity describes the retention of both hydrophobic and hydrophilic nature in a protein. Six conserved peaks of hydrophobicity correlate with six peaks of similarity, which correspond to six TMDs among AtRbohs and OsRbohs. All these peaks displayed moderate level of amphipathicity. The peaks of amphipathicity in loops between TMDs exceeded the amphipathicities of the six TMDs within 19 Rbohs. Among OsRbohs, a large insertion in TMD-III of OsRbohF showed low similarity. To show that TMD-III is well-conserved, average hydropathy, amphipathicity and similarity plot was constructed by removing OsRbohF (Figure 4c). In addition, one peak of hydrophobicity, similarity and amphipathicity was observed within AtRbohs and OsRbohs. The results also showed that Nterminal had least similarity among AtRbohs and OsRbohs. Also, it appeared more hydrophilic as compared to C-terminal. There was no clear peak of amphipathicity corresponding to the Nterminal among AtRbohs and OsRbohs.

## Discussion

In the present work, we were focussed on in silico physiochemical characterization of 19 Rboh proteins (10 from A. thaliana and 9 from O. sativa Japonica), their topological analysis, subcellular localization and signal peptide detection. The most fundamental characteristics of protein sequences are length and size (molecular weight). In our study, more variation in protein length and molecular weights was observed in rice Rbohs as compared to Arabidopsis. The isoelectric point (pI) and charge are also important parameters for solubility, subcellular localization and interaction. The pI denotes the pH value at which the protein carries no charges or the negative and positive charges are equal. It was observed that the calculated pI was > 7 for 19 Rbohs which indicates their basic nature. The basic nature and large size of these transmembrane proteins is consistent with the previous report inferring membrane proteins as heavier and more basic than non-membrane proteins in bacteria, archaea and eukaryotes [20, 21]. These observations are also in agreement with the view that membrane bilayer is negatively charged and basic amino acids from these proteins have proper electrostatic interactions, which promote their stability in the membrane. In addition, transmembrane proteins are evolving rapidly to adjust with the external environment so that they can interact with an extensive range of partners. Also, for the purification of a protein by isoelectric focusing methods, the pI value will be useful for developing buffer system. In addition to pI, the instability index (II) provides an estimation of the stability of the protein in vitro and in vivo. A protein whose instability index is <40 indicates stable and the value >40 infers unstable protein [22] . The lowest instability index observed for AtRbohD indicated its stability and hence its ability to play multiple roles in plant development, biotic and abiotic stress conditions [1]. Similarly, other well studied Rbohs found to possess instability index below 40 were AtRbohB and OsRbohB with 38.55 and 39.76, respectively. AtRbohB is involved in seed germination and after-ripening [23] while OsRbohB is the only plant Rboh which has been crystallized [3] and also involved in immune response [24]. Another measure for stability of proteins is the aliphatic index (AI) and increase in its value is reported to enhance the thermo stability of globular proteins [25]. AI refers to the relative volume occupied by aliphatic side chain of the following amino acids: alanine (A), isoleucine (I), leucine (L) and valine (V). The lowest AI of OsRbohF is indicative of its low thermal stability and hence of more flexible structure when compared to other Rbohs. The high AI of OsRbohA, AtRbohB, OsRbohC, AtRbohG, AtRbohJ and AtRbohE inferred that Rbohs might be stable under a wide range of temperature conditions. Further analysis of amino acid percentage composition revealed leucine to be the most abundant amino acid among AtRbohs and OsRbohs. This observation is consistent with an earlier report documenting the high occurrence of leucine in membrane proteins [21]. Also, our pattern of amino acid frequencies correlate with that of earlier report on membrane proteins [21]. In addition, extinction coefficient of Rbohs was also computed at 280 nm. The calculated ECs of Rbohs indicated the presence of high concentration of tyrosine (Y) and tryptophan (W), and not of cysteine (C) because it was observed in very low amount in all Rbohs. This indicated that UV spectral methods couldn't be employed to analyze Rbohs. However, the obtained EC values will aid in the study of protein-protein and protein-ligand interactions [26].

Similar to stability and protein concentration, it is also critical to evaluate the hydrophobic or hydrophilic character and topology of the protein. For this purpose, GRAVY score and topology analysis were done. GRAVY score denotes the sum of hydropathy values of all amino acids in the protein, divided by the number of residues in the protein. It lies in the range from -2 to +2 where positive value represents hydrophobic and negative indicates hydrophilic protein [27]. It is also an indicator of whether a protein would be observed on 2-D gels, as proteins having GRAVY scores >0.4 does not lie in solubility range and hence are difficult to detect [28]. In case of Rbohs, GRAVY score exhibited a very narrow range (-0.087 to -0.286) with less negative value indicating a low hydrophobic nature and hence good solubility. This may be due to the presence of hydrophilic Nterminal and six hydrophobic TMDs, which is further in agreement with our topological analysis. These lines of evidence are also consistent with earlier studies reporting hydrophilic proteins with TMDs [29, 30] as well as six TMDs in Rbohs [31, 32]. In addition to 6 TMDs, topological analysis also revealed a separate hydrophobic peak, which indicate the conserved glycine-rich motif (GXGXG) from NADPH binding domain of Rbohs. The glycine-rich motif has been reported in substrate binding, where substrate could be ATP and S-adenosyl-Lmethionine (SAM) in histidine kinases and SAM-dependent methyltransferases, respectively [33, 34]. Other kind of glycinerich motif (GXXXG) is documented in transmembrane α-helices and help in stabilizing the oligomerization of membrane proteins [35].

## Conclusion

The current study sheds light on the variations in the vital properties such as molecular weight, isoelectric point, and total number of negatively and positively charged residues, extinction coefficient, instability index, aliphatic index and grand average of hydropathicity within Rbohs proteins, which may be responsible for their functional multiplicity. Insights from the evaluation of their hydrophobic or hydrophilic character and topology are also reported.

## Conflict of Interest

Authors declare no conflict of interest.

## Supplementary material

S1 File

## Figures and Tables

**Table 1:** Rboh protein sequences retrieved from UniProt

Species	Protein name	Accession No.
Arabidopsis thaliana	AtRbohA	O81209
	AtRbohB	Q9SBI0
	AtRbohC	O81210
	AtRbohD	Q9FIJ0
	AtRbohE	O81211
	AtRbohF	O48538
	AtRbohG	Q9SW17
	AtRbohH	Q9FJD6
	AtRbohI	Q9SUT8
	AtRbohJ	Q9LZU9
Oryza sativa	OsRbohA	Q0JJJ9
	OsRbohB	Q5ZAJ0
	OsRbohC	Q65XC8
	OsRbohD	Q0DHH6
	OsRbohE	Q8S1T0
	OsRbohF	Q0J595
	OsRbohG	Q69LJ7
	OsRbohH	Q2QP56
	OsRbohI	Q2R351

**Table 2:** Physicochemical properties of 19 Rboh proteins computed using ExPASy ProtParam tool.

Rbohs	Length	M. wt.	pI	(-) R	(+)R	ε, 280 (in -1 cm-1)	II	AI	GRAVY
AtRbohA	902	102935.4	9.26	93	115	150745	45.57	85.24	-0.229
AtRbohB	843	96390.1	9.26	85	107	147305	38.55	89.37	-0.16
AtRbohC	905	102518.2	9.5	86	117	143295	42.59	86.08	-0.219
AtRbohD	921	103908.6	9.27	99	122	152345	38.32	86.21	-0.241
AtRbohE	952	107702.7	8.95	106	123	145520	44.03	87.07	-0.207
AtRbohF	944	108418.2	9.23	100	121	164600	47.17	85.73	-0.276
AtRbohG	849	96862.4	9.1	90	109	143295	40.43	87.75	-0.196
AtRbohH	886	100627.5	9.25	87	111	144910	42.45	83.88	-0.201
AtRbohI	941	106952	8.71	95	106	162120	48.99	85.26	-0.229
AtRbohJ	912	102936.9	9.48	82	114	146275	44.05	87.21	-0.203
OsRbohA	743	85152.8	8.98	76	90	140900	45.31	93.2	-0.087
OsRbohB	905	101758.8	9.33	95	119	145355	39.76	82.31	-0.254
OsRbohC	951	107171.3	9.35	97	122	151775	50.23	87.82	-0.217
OsRbohD	819	92349.5	9.18	78	97	121990	46.91	85.4	-0.123
OsRbohE	843	94790.1	9.38	81	105	117855	44.05	82.28	-0.211
OsRbohF	1033	115014.5	9.84	95	135	178815	52.79	77.51	-0.286
OsRbohG	1007	112134	9.46	97	119	165170	49.34	81.84	-0.204
OsRbohH	909	102122.9	9.2	90	108	157970	46.06	82.73	-0.205
OsRbohI	936	105185	9.24	102	122	156940	45.84	82.54	-0.272
M. wt., pI , (-) R, (+) R, (ε, 280), II, AI and GRAVY denotes molecular weight, isoelectric point, total number of negatively charged residues, total number of positively charged residues, extinction coefficient at 280 nm, instability index, aliphatic index and grand average of hydropathicity.									

**Table 3:** Amino acid composition of 19 Rboh proteins (in percentage) computed using ExPASy ProtParam tool.

	A	R	N	D	C	Q	E	G	H	I	L	K	M	F	P	S	T	W	Y	V
AtRbohA	6.2	7	4.3	4.6	1.2	2.2	5.7	6	2.8	5.2	9.2	5.8	3.3	4.7	4.1	8.3	5.3	1.9	4.2	7.9
AtRbohB	6.2	5.9	5.3	4.3	1.3	2.4	5.8	5.9	2.4	6	9.7	6.8	2.6	5.7	2.8	7.7	5.7	2.1	3.8	7.5
AtRbohC	7.5	6.7	4.5	4.5	1.2	2.8	4.8	6.4	2.8	5	9.5	6.2	2.9	4.8	4.5	6.5	5.9	1.9	3.6	7.6
AtRbohD	7.6	5.6	4.8	5.9	1.1	2.7	4.8	6.9	2	5.9	9.8	7.6	2.8	5.2	3.9	7.2	4.7	2.2	3	6.1
AtRbohE	6.8	6.8	3.3	5.9	2.1	3	5.3	5.6	2.3	5.4	10.6	6.1	2.5	4.7	3	9	6.7	2.1	2.4	6.2
AtRbohF	6.1	6.6	4.2	5.1	1	4	5.5	5.9	2	6.2	9.7	6.2	2.2	5.8	3.2	8.2	5.7	2	4.2	5.9
AtRbohG	6.9	4.4	4.5	4.5	1.3	2.9	6.1	5.4	2.2	5.9	10	8.5	3.7	4.8	4.2	6.6	5.5	2	3.9	6.5
AtRbohH	6.9	5.4	4.5	3.6	1.5	2.5	6.2	6.7	2.4	5.8	9.7	7.1	3.7	5.3	4.4	7.2	5.5	1.9	3.8	5.8
AtRbohI	5.3	6.1	4.5	4.8	1.8	3	5.2	6.1	2.9	6.6	9.6	5.2	2	5.2	3.7	10.4	5.7	2	4	5.8
AtRbohJ	5.9	5.7	4.8	3.5	1.2	2.9	5.5	7.3	2.4	6.4	9.4	6.8	3.1	4.5	4.5	8.2	5	1.9	3.8	6.8
OsRbohA	7	5.7	3.5	4.5	1.6	2.8	5.7	5.8	2.3	7.1	10.8	6.5	2.8	5.1	3.9	6.3	6.1	2.2	4.6	5.7
OsRbohB	8	6	3.4	5.2	1.1	3.5	5.3	6.9	2.3	4.1	9.1	7.2	2.5	5.2	3.8	7.5	6	2.1	3	8
OsRbohC	8.2	7.3	2.9	4.5	1.2	3.5	5.7	6.7	2	6.2	10	5.6	2.3	4.4	4.5	7.7	6	1.9	3.7	5.7
OsRbohD	8.1	5.5	3.7	4.8	1.6	2.7	4.5	5.7	3.1	5.4	9.6	6.3	2.9	5.9	4.5	7.8	6.2	1.8	3.2	6.5
OsRbohE	8.3	6.4	4	4.2	1.3	2.5	5.5	7.1	3.3	5	9.1	6	3.1	5.5	4.5	7	5.7	1.7	3.2	6.5
OsRbohF	10.1	9.3	2.5	3.8	1.1	2.6	5.4	8	2.1	3.5	9.8	3.8	2.5	4.5	5.6	8.8	5.7	2.3	3	5.4
OsRbohG	10.9	8.5	3.3	3.9	0.8	3	5.8	7.7	2.2	3.6	10	3.3	2.6	4.8	4.6	7.2	6.2	2.1	3.3	6.2
OsRbohH	8.1	7.7	3.5	5.2	1.4	2.9	4.6	8.1	3.1	5	9	4.2	2.6	5.2	4.5	6.8	5.5	2.3	3.1	6.9
OsRbohI	9.1	8.1	3.7	5.7	1.3	2.6	5.2	7.5	2.6	4.2	9.2	4.8	2	5	4.4	7.2	4.5	2.1	3.3	7.4

## References

[1] Kaur G (2014). Biotechnol Adv.

[2] Groom QJ (1996). Plant J.

[3] Oda T (2010). J Biol Chem.

[4] Kaur G (2015). Agriculture Bioinformatics.

[5] Lee D (2007). Nat Rev Mol Cell Biol.

[6] Kaur G (2018). Amino Acids.

[7] Kaur G, Pati PK. (2016). Comput Biol Chem.

[8] Gasteiger E (2005). The proteomics protocols handbook.

[9] Horton P (2007). Nucleic Acids Res.

[10] Yu CS (2006). Proteins.

[11] Chang TH (2013). J Comput Aided Mol Des.

[12] Petersen TN (2011). Nat Methods.

[13] Hiller K (2004). Nucleic Acids Res.

[14] Krogh A (2001). J Mol Biol.

[15] Käll L (2004). J Mol Biol.

[16] Tusnády GE, Simon I. (2001). Bioinformatics.

[17] Zhai Y, Saier MH. (2001). J Mol Micro Biotechnol.

[18] Thompson JD (1997). Nucleic Acids Res.

[19] Zhai Y, Saier Jr MH. (2001). J Mol Micro Biotechnol.

[20] Knight CG (2004). Proc Natl Acad Sci USA.

[21] Schwartz R (2001). Genome Res.

[22] Guruprasad K (1990). Protein Eng.

[23] Müller K (2009). New Phytol.

[24] Li Y (2011). Chinese J Biotechnol.

[25] Atsushi I. (1980). J Biochem.

[26] Gill SC, Von Hippel PH. (1989). Anal Biochem.

[27] Kyte J, Doolittle RF. (1982). J Mol Biol.

[28] Wilkins MR (1998). Electrophoresis.

[29] Fountoulakis M, Gasser R. (2003). Amino Acids.

[30] Zhang L (2005). Proteomics.

[31] Lin F (2009). J Integr Plant Biol.

[32] Trujillo M (2006). J Exp Bot.

[33] Egger LA (1997). Genes Cells.

[34] Kozbial PZ, Mushegian AR. (2005). BMC Struct Biol.

[35] Kleiger G, Eisenberg D. (2002). J Mol Biol.

