# Supplementary material for: In silico physicochemical characterization and topology analysis of Respiratory burst oxidase homolog (Rboh) proteins from Arabidopsis and rice
Source: Bioinformation. 2018 Mar 31;14(3):93–100. doi: 10.6026/97320630014093 (PMC5953861; doi:10.6026/97320630014093)
Supplement: S1 File [file 97320630014093S1.pdf]

```
OsRbohA_Q0JJJ9      ------------------------------------------------------------
OsRbohB_Q5ZAJ0      -----------M--ADLEAGMVAAAT------------------------------DQGN 17
OsRbohC_Q65XC8      -----MRAGIGSG----------------------SGGGTTPVRPRWGSGVTT----PR- 28
OsRbohD_Q0DHH6      ------------------------------------------------------------
OsRbohE_Q8S1T0      ------------------------------------------------------------
OsRbohF_Q0J595      -MWTPSRGSASGRRATGHRRIADYLADDRTE--A--STENGSFNTAYSDELFAPTSSSAG 55
OsRbohG_Q69LJ7      -MWTPSRGSNAAR-RSGHRRIADYLADDQTT--NTDTSDNESYTTAYGDEFFAAAAA-AA 55
OsRbohH_Q2QP56      -MASREESGNG-------------------------------------------G---- 11
OsRbohI_Q2R351      MHHTRAGAADGA--GGGGEDIVEAGAEAPPPQRERLVP--------HSGPLSKRS-GMRK 49
AtRbohA_O81209      -MMNR-----------------------SEMQKLGFEHVRY----YTESP---------- 22
AtRbohB_Q9SBI0      ---------------------------------------------------------MRE 3
AtRbohC_O81210      -----------------------------MSRVSFEVSGG----YHSDAEAGNSGPM-- 24
AtRbohD_Q9FIJ0      MKMRRGNSSND-----HELGILRGANSDTNSDTESIASDRG----AFSGPLGRPKRASKK 51
AtRbohE_O81211      MKLSPLSFSTSSS----------------------FSHADG---IDDGVELIS----SP- 30
AtRbohF_O48538      -----------------------------------MKPFSKNDRRRWSFDSVS----AGK 21
AtRbohG_Q9SW17      ------------------------------MQRVSFEVKDT----EA----EKSSSEI-- 20
AtRbohH_Q9FJD6      ------------------------------------------------------------
AtRbohI_Q9SUT8      ---------MSMS----------------------FSG--GTHNDRWGSDLAS----AGE 23
AtRbohJ_Q9LZU9      ------------------------------------------------------------


OsRbohA_Q0JJJ9      ------------------------------------------------------------
OsRbohB_Q5ZAJ0      STRSQDDA-ATLIPNSGNLGSSNRSTKTARFKDDDELVEITLDVQRDS-VAIQE-VRGVD 74
OsRbohC_Q65XC8      -SLSTGS---------------SPRGSDRSSDDGEELVEVTLDLQEDDTIVLRS-VEPAA 71
OsRbohD_Q0DHH6      ------------------------------------------------------------
OsRbohE_Q8S1T0      ------------------------------------------------------------
OsRbohF_Q0J595      GDGVGGMLPA-FLAD------------------QSDLVEVMLELDEESMVV-RS-VTPTT 94
OsRbohG_Q69LJ7      GSGGGGMLPA-FLAD------------------QGDLVEVMLELDEESMVV-RS-VTPTS 94
OsRbohH_Q2QP56      ---GGGATPAADYRSSD---SRSSSRRSTRFKEDNEYVEITLDVKGDDTVAIQS-IRN-- 62
OsRbohI_Q2R351      SARFAESV-SAPLSAPSGVAAARRAAAAANDDDEEDYVEITLDVRDDS-VAVHS-VKPAG 106
AtRbohA_O81209      -----------YNRGES---SANVATTSNYYGEDEPYVEITLDIHDDS-VSVYG-LKSPN 66
AtRbohB_Q9SBI0      E-----EM---ESSSEGETNKISRCKATGSDNPDEDYVEITLEVRDET-INTMK-AKAT- 52
AtRbohC_O81210      ---SGGQL-PPIYKKPG---NSRFTAENSQRTRTAPYVDLTVDVQDDT-VSVHS-LKMEG 75
AtRbohD_Q9FIJ0      NARFADDL-P-----------KRSNSVAGGRGDDDEYVEITLDIRDDS-VAVHS-VQQAA 97
AtRbohE_O81211      -FAGGAMLPV-FLNDL----SRNSGESGSGSSWERELVEVTLELDVGDDSILVCGMSEAA 84
AtRbohF_O48538      TAVGSAS---------------TSPGTEYSINGDQEFVEVTIDLQDDDTIVLRS-VEPAT 65
AtRbohG_Q9SW17      ---LSGSL-PSTYRNPA---MENV-----------------------------G-NAVDD 43
AtRbohH_Q9FJD6      --------------------MKSNTPTEDSTKWMLESVEI-------------------- 20
AtRbohI_Q9SUT8      FTQSFPSLPA-----------TYSPSPSSSSSSGEELLEVTIEFPSGVIINIDS-VTGTG 71
AtRbohJ_Q9LZU9      --------------MK----NNKKVGTEDSTKWMLESVEI-------------------- 22

OsRbohA_Q0JJJ9      ------------------------------------------------------------
OsRbohB_Q5ZAJ0      EGGSGHGTGFDGL-PLVSPSSKS------G--------------------------KLTS 101
OsRbohC_Q65XC8      GGAAVASSSGASPSAVAPPRRAEPPGG-VASR-SRSPAMRRTSSHRLLQFSQELKAEAMA 129
OsRbohD_Q0DHH6      ---MAGD-------------YVDVPLGGGGQSTL-------------------------- 18
OsRbohE_Q8S1T0      ---MASPYDHQSPHAQHPSGLPRPPGAGAGAAAG-------------------------- 31
OsRbohF_Q0J595      G-ALYGPTSLAGG------GAAHTPPGSGRSL-SRCS----S------------------ 124
OsRbohG_Q69LJ7      A-TLYGGGGGQMPQPLPPPLRTPEGGGGARSL-SRCS----S------------------ 130
OsRbohH_Q2QP56      ------GADMPEV-ALLARGLAQ------QPP-P-------SAAPGPG--------GLSS 93
OsRbohI_Q2R351      GGGE--DSDV----TLLART-LE------KR--SS-----SFGHSVIR--------NASS 138
AtRbohA_O81209      HRGA--GSNYEDQ-SLLRQGRSG------RSN-SV-----LK--------------RL-- 95
AtRbohB_Q9SBI0      ---------------------LR------SVL-S-------------------------G 59
AtRbohC_O81210      GSSV--EES-PEL-TLLKRNRLE------KKT-TV-----VK--------------RL-- 103
AtRbohD_Q9FIJ0      GGGG--HLEDPEL-ALLTKKTLE------SSL-NN-----TTSLSFFR--------STSS 134
AtRbohE_O81211      S--------------------------VDSR-ARSVDLVTA------RLSRNLS-NAST 109
AtRbohF_O48538      AINVIGDISDDNTGIMT-----------PVSI-SRSPTMKRTSSNRFRQFSQELKAEAVA 113
AtRbohG_Q9SW17      GSSV--KNN-PKL-DMQKQNGLV------KWF-K-------------------------- 66
AtRbohH_Q9FJD6      --DSMGESSSKEPEINLNKN-------EGGLKKNASRNLGVG------------------ 53
AtRbohI_Q9SUT8      T-----DISGTDLEITSC---S--DSG-SG---SRSLSLGWS------ASSERLTA--GT 109
AtRbohJ_Q9LZU9      --DPKGDSSVKQPESTINSNNPESSGAGGGILKNVSKNLAVG------------------ 62
```

```
OsRbohA_Q0JJJ9    ------------------------------------------------------------
OsRbohB_Q5ZAJ0    KLRQVTNGLKMKSSSRKAP-----------------------SPQAQQSAKRVRKRLDRT 138
OsRbohC_Q65XC8    RARQFSQ-DLTKRF-----TRT---------QSTTTAPPGIESALAARAERRQRAQLDRT 174
OsRbohD_Q0DHH6    ----PPVAPLKKQP----SRLASGMK---RLAS----------------MVPDTMKLKRT 51
OsRbohE_Q8S1T0    ----GFARGLMKQP----SRLASGVR---QFASRVSMKVP-E---GVGGMRPGGGRMTRM 76
OsRbohF_Q0J595    ----TSSRIRKKFAWLRSPSPAP----APRAPTPSEPPPPREAAMAARERRRIQARLNRS 176
OsRbohG_Q69LJ7    ----TSSRIRKKFAWLRSPSPSP----SPRPPTPAEL--QREAAMAARERRRIQARLNRS 180
OsRbohH_Q2QP56    RLKAVRTELRRIASWKFPSGVLSG---------------GGGGGDAPGNGNDRRPRLDRS 138
OsRbohI_Q2R351    RIKQVSQELRRLASV------------------------------NRRGGGGGGPRFDRS 168
AtRbohA_O81209    -ASSVSTGITRVASSVS-------------------------SSSARKPPRPQLAKLRRS 129
AtRbohB_Q9SBI0    RLKTMV-----------------------------------------KSLSFASRRLDRS 78
AtRbohC_O81210    -AS-VSHELKRLTSVSG-------------------------GIGGRKPPR--PAKLDRT 134
AtRbohD_Q9FIJ0    RIKNASRELRRVFS---------------------------------RRPSPAVRRFDRT 161
AtRbohE_O81211    RIRQKLGKLLRSESWKTTTSST----AGERDRDLERQTAVTLGILTARDKRKEDAKLQRS 165
AtRbohF_O48538    KAKQLSQ-ELKRFSWSRSFSGNLTTTSTAANQSGGAGGGLVNSALEARALRKQRAQLDRT 172
AtRbohG_Q9SW17    ---------KCLTMVSG-------------------------------ESK--APRLDRS 84
AtRbohH_Q9FJD6    ----SIIRTLSVSNWRKSGNLGSPST---RKSGN--LGPPTN---AV-PKKTGPQRVERT 100
AtRbohI_Q9SUT8    NSKQQIQ-KISRRGYGYSSRS-------APEPVVPHRGEITDSVNLPRALSQRPTRPNRD 161
AtRbohJ_Q9LZU9    ----SIIRSMSVNKWRKSGNLGSPST---RKSGN--LGPPLP---VSQVKRPGPQRVERT 110

                                                                EF-hand-like I
OsRbohA_Q0JJJ9    ----------------------------------------------AR--DGYLSRSDFA 12
OsRbohB_Q5ZAJ0    KS-SAAVALKGLQFVTAKV-GN-----------DGWAAVEKRFNQIQV--DGVLLRSRFG 183
OsRbohC_Q65XC8    KS-GAQRAIRGLRFISGPNKAS-----------NAWIEVQANFDRIAR--DGYLSRDDFP 220
OsRbohD_Q0DHH6    HS-SAQPALRGLRFLDKTSAG-----------KDGWKNVEKRFDEMSA--DGRLPQESFA 97
OsRbohE_Q8S1T0    QS-SAQVGLRGLRFLDKTSGG-----------KEGWKSVERRFDEMNR--NGRLPKESFG 122
OsRbohF_Q0J595    RS-GARRALKGLRFISRTTGSA--------EAAELWTRVEHRFNAISR--DGLLSRDNFG 225
OsRbohG_Q69LJ7    ST-GAKRALKGLRFISRTTGTV--------QAAELWRRVEDRFNAIAR--DGLLSRDDFG 229
OsRbohH_Q2QP56    MT-GAARALRGLQFLNSSA-VT-----------NGWPEVEKRFERIAV--DGFLLRSRFG 183
OsRbohI_Q2R351    KS-AAAHALKGLKFISRAD-GG-----------AGWPAVEKRFDDIAK--DGLLPRSKFG 213
AtRbohA_O81209    KS-RAELALKGLKFITKTD-GV-----------TGWPEVEKRFYVMTMTNNGLLHRSRFG 176
AtRbohB_Q9SBI0    KSFGAMFALRGLRFIAKNDAVG-----------RGWDEVAMRFDKIAV--EGKLPKSKFG 125
AtRbohC_O81210    KS-AASQALKGLKFISKTD-GG-----------AGWSAVEKRFNQITATTGGLLLRTKFG 181
AtRbohD_Q9FIJ0    SS-AAIHALKGLKFIAT---KT-----------AAWPAVDQRFDKISADSNGLLLSAKFW 206
AtRbohE_O81211    TS-SAQRALKGLQFINKTTRGNSCVCDWDCDCDQMWKKVEKRFESISK--NGLLARDDFG 222
AtRbohF_O48538    RS-SAQRALRGLRFISNKQKNV-----------DGWNDVQSNFEKEEK--NGYIYRSDFA 218
AtRbohG_Q9SW17    KS-TAGQALKGLKIISKTD-GN-----------AAWTVVEKRYLKITANTDGLLLRSKFG 131
AtRbohH_Q9FJD6    TS-SAARGLQSLRFLDRTVTGR---------ERDAWRSIENRFNQESV--DGKLPKEKFG 148
AtRbohI_Q9SUT8    GS-GTERAIHGLKFISSKENGI-----------VDWNDVQNNFAHISK--DGYLFKSDFA 207
AtRbohJ_Q9LZU9    TS-SAARGLQSLRFLDRTVTGR---------ERDSWRSIENRFNQEAV--DGRLPKDKFG 158

                                                                      EF-hand-like II
OsRbohA_Q0JJJ9    ECIGMT-------------------------------ESKEFALELFDTLSRRRQMKVD- 40
OsRbohB_Q5ZAJ0    KCIGMD------------------------------G-SDEFAVQMFDSLARKRGIVKQ- 211
OsRbohC_Q65XC8    QCIGMT-------------------------------ESKEFAMELFDTLSRRRQMQVD- 248
OsRbohD_Q0DHH6    KCIGMA-------------------------------DSKEFASEVFVALARRRSIKPED 126
OsRbohE_Q8S1T0    KCIGMG-------------------------------DSKEFAGELFVALARRRNLEPED 151
OsRbohF_Q0J595    DCIGKQAKPSSMSMARRARARSRDDTAYGAGIGAGMEDSKEFAVGIFDALARRRRQELE- 284
OsRbohG_Q69LJ7    ECIG-------------------------------MVDSKEFAVGIFDALARRRRQNLE- 257
OsRbohH_Q2QP56    QCIGMV------------------------------G-SEEFAVQIFDSLARRRGITAQ- 211
OsRbohI_Q2R351    QCIGMK------------------------------E--LEFAGELFDALARRRNISGD- 240
AtRbohA_O81209    ECIGMK------------------------------S--TEFALALFDALARRENVSGD- 203
AtRbohB_Q9SBI0    HCIGMV------------------------------E-SSEFVNELFEALVRRRGTTSS- 153
AtRbohC_O81210    ECIGMT------------------------------S--KDFALELFDALARRRNITGE- 208
AtRbohD_Q9FIJ0    ECLGMN------------------------------KESKDFADQLFRALARRNNVSGD- 235
AtRbohE_O81211    ECVGMV-------------------------------DSKDFAVSVFDALARRRRQKLE- 250
AtRbohF_O48538    QCIGMK-------------------------------DSKEFALELFDALSRRRRLKVE- 246
AtRbohG_Q9SW17    ECIGMN------------------------------S--KEFALELFDALARKSHLKGD- 158
AtRbohH_Q9FJD6    VCIGMG-------------------------------DTMEFAAEVYEALGRRRQIETEN 177
AtRbohI_Q9SUT8    HCIGLE-----------------------------NENSKEFADELFDALCRRRRIMVD- 237
AtRbohJ_Q9LZU9    VCIGMG-------------------------------DTLEFAAKVYEALGRRRQIKTEN 187
```

```
                         EF-hand-like II               EF-hand I
OsRbohA_Q0JJJ9     TINKDELREIWQQITDNSFDSRLQIFFEMVDKNADGRITEAEVKEIIMLSASANKLSRLK 100
OsRbohB_Q5ZAJ0     VLTKDELKDFYEQLTDQGFDNRLRTFFDMVDKNADGRLTAEEVKEIIALSASANKLSKIK 271
OsRbohC_Q65XC8     KINKEELREIWQQITDNSFDSRLQIFFDMVDKNADGHITEAEVKEIIMLSASANKLSRLK 308
OsRbohD_Q0DHH6     GITKEQLKEFWEELTDQNFDSRLRIFFDMCDKNGDGQLTEDEVKEVIVLSAAANKLAKLK 186
OsRbohE_Q8S1T0     GITKEQLKEFWEEMTDQNFDSRLRIFFDMCDKNGDGMLTEDEVKEVIILSASANKLAKLK 211
OsRbohF_Q0J595     RISKEELYDFWLI--------------------------------VLSASANKLSKLK 310
OsRbohG_Q69LJ7     RITREELYDFWLQISDQSFDARLQIFFDMVDTNVDGRITREEVQELIVLSASANKLAKLK 317
OsRbohH_Q2QP56     LLTKDQLREFWEQLSDPGFDAKLQTFFDMVDKNADGQITEEELKEVLTLTASANKLSKIL 271
OsRbohI_Q2R351     SISKAELLEFWDQISDTSFDSRLQTFFDMVDKNADGRITEEEVKEIITLSASANKLSKVQ 300
AtRbohA_O81209     SININELKEFWKQITDQDFDSRLRTFFAMVDKDSDGRLNEAEVREIITLSASANELDNIR 263
AtRbohB_Q9SBI0     SITKTELFEFWEQITGNSFDDRLQIFFDMVDKNLDGRITGDEVKEIIALSASANKLSKIK 213
AtRbohC_O81210     VIDGDQLKEFWEQINDQSFDSRLKTFFDMVDKDADGRLTEDEVREIISLSASANNLSTIQ 268
AtRbohD_Q9FIJ0     AITKEQLRIFWEQISDESFDAKLQVFFDMVDKDEDGRVTEEEVAEIISLSASANKLSNIQ 295
AtRbohE_O81211     KITKDELHDFWLQISDQSFDARLQIFFDMADSNEDGKITREEIKELLMLSASANKLAKLK 310
AtRbohF_O48538     KINHDELYEYWSQINDESFDSRLQIFFDIVDKNEDGRITEEEVKEIIMLSASANKLSRLK 306
AtRbohG_Q9SW17     VITETELKKFWEQINDKSFDSRLITFFDLMDKDSDGRLTEDEVREIIKLSSSANHLSCIQ 218
AtRbohH_Q9FJD6     GIDKEQLKLFWEDMIKKDLDCRLQIFFDMCDKNGDGKLTEEEVKEVIVLSASANRLGNLK 237
AtRbohI_Q9SUT8     KINLQELYEFWYQITDESFDSRLQIFFNMV-KNGDGRITENEVKEIIILSASANNLSRLR 296
AtRbohJ_Q9LZU9     GIDKEQLKLFWEDMIKKDLDCRLQIFFDMCDKDGDGKLTEEEVKEVIVLSASANRLVNLK 247


                                 EF-hand II
OsRbohA_Q0JJJ9     EQAEEYAALIMEELDPEGL--GYIE-----------------------LWQLETLLLQKD 135
OsRbohB_Q5ZAJ0     ERADEYTALIMEELDPTNL--GYIE-----------------------MEDLEALLLQSP 306
OsRbohC_Q65XC8     EQAEEYAALIMEELDPEQL--GYIE-----------------------LWQLETLLLQKD 343
OsRbohD_Q0DHH6     SHAATYASLIMEELDPDHR--GYIE-----------------------IWQLETLLRGMV 221
OsRbohE_Q8S1T0     GHAATYASLIMEELDPDDR--GYIE-----------------------IWQLETLLRGMV 246
OsRbohF_Q0J595     EQAEEYASLIMEELDPEDL--GYIE-----------------------LWQLEALLLQRD 345
OsRbohG_Q69LJ7     EQAEEYASLIMEELDPENL--GYIE-----------------------LWQLEALLLQRD 352
OsRbohH_Q2QP56     ERVDEYTALIMEELDPDQL--GYID-----------------------ISNLESLLLLPP 306
OsRbohI_Q2R351     EQSEEYARLIMEELDPSNL--GYIE-----------------------LYNLEMLLLQAP 335
AtRbohA_O81209     RQADEYAALIMEELDPYHY--GYIM-----------------------IENLEILLLQAP 298
AtRbohB_Q9SBI0     ENVDEYAALIMEELDRDNL--GYIE-----------------------LHNLETLLLQVP 248
AtRbohC_O81210     KRADEYAALIMEELDPDNI--GYIM-----------------------LESLETLLLQAA 303
AtRbohD_Q9FIJ0     KQAKEYAALIMEELDPDNA--GFIM-----------------------IENLEMLLLQAP 330
AtRbohE_O81211     EQAEEYASLIMEELDPENF--GYIE-----------------------LWQLETLLLQRD 345
AtRbohF_O48538     EQAEEYAALIMEELDPERL--GYIE-----------------------LWQLETLLLQKD 341
AtRbohG_Q9SW17     NKADEYAAMIMEELDPDHM--GYIM-----------------------MESLKKLLLQAE 253
AtRbohH_Q9FJD6     KNAAAYASLIMEELDPDHK--GYIE-----------------------MWQLEILLTGMV 272
AtRbohI_Q9SUT8     ERAEEYAALIMEELAPDGLYSQYIE-----------------------LKDLEILLLEKD 333
AtRbohJ_Q9LZU9     KNAASYASLIMEELDPNEQ--GYIE-----------------------MWQLEVLLTGIV 282


OsRbohA_Q0JJJ9     TYMNYSQALSYTSQAL-SQNLA--------------------------------GL-RKK 161
OsRbohB_Q5ZAJ0     SEAAARS-TTTHSSKL-SKALS-----------------------------MKLASNKEM 335
OsRbohC_Q65XC8     TYVNYSQALSYTSQAL-SQNLA--------------------------------GL-RKR 369
OsRbohD_Q0DHH6     TAQGPPEKVKLASASL-----AR-----------------------------TMVPSSHR 247
OsRbohE_Q8S1T0     SAQAAPEKMKRTTSSL-----AR-----------------------------TMIPSRYR 272
OsRbohF_Q0J595     AYMNYSRPLSSGSTAQWSQNLGGGGGGQQG-----------GQGQGQGQSEGRRNDWRRR 394
OsRbohG_Q69LJ7     SYMNYSRPLSTASGAQWSQNLGGAAVAAGAAAATGGGAHAAVAARGGQQQQQQQEGRRGG 412
OsRbohH_Q2QP56     SQAPSKL--VTHSSNI-SQLIS----------------------------QKLVPTHDR 334
OsRbohI_Q2R351     SQSVRI--GTTNSRNL-SQMLS----------------------------QNLRPTAEP 363
AtRbohA_O81209     MQD----VRDGESKKL-SKMLS----------------------------QNLMVPQSR 324
AtRbohB_Q9SBI0     SQSNNSP-SSANKRAL-NKMLS----------------------------QKLIPTKDR 277
AtRbohC_O81210     TQSVITS--TGERKNL-SHMMS----------------------------QRLKPTFNR 331
AtRbohD_Q9FIJ0     NQSVRM--GD--SRIL-SQMLS----------------------------QKLRPAKES 356
AtRbohE_O81211     AYMNYSRPLSTTSGGV-STPRR--------------------------------NLIRPR 372
AtRbohF_O48538     TYLNYSQALSYTSQAL-SQNLQ--------------------------------GL-RGK 367
AtRbohG_Q9SW17     TKSVSTDINSEERKEL-SDMLT----------------------------ESLKPTRDP 283
AtRbohH_Q9FJD6     TNAD-TEK-MKKSQTL-----TR-----------------------------AMIPERYR 296
AtRbohI_Q9SUT8     ISHSYSLPFSQTSRAL-SQNLK--------------------------------DR-R-- 357
AtRbohJ_Q9LZU9     SNAD-SHKVVRKSQQL-----TR-----------------------------AMIPKRYR 307
```

```
                                              TMD-I           A-loop
OsRbohA_Q0JJJ9      SSIRKISTSLSYYFEDNWKFLWVLALWIGIMAGLFTWKFMQYRNRYVFDVMGYCVTTAKG 221
OsRbohB_Q5ZAJ0      SPVRHYWQQFMYFLEENWKFSWVMTLWISICIALFIWKFIQYRNRAVFGIMGYCVTTAKG 395
OsRbohC_Q65XC8      SPIRKISTKLSYYLEDNWKFLWVLALWIGIMAGLFIWKFIQYRHRYVFNVMGYCVTTAKG 429
OsRbohD_Q0DHH6      SPMQRRFNKTVDFIHENWKFIWVLSLWAILNIALFMYKFVQYSRRDAFQVMGYCVCIAKG 307
OsRbohE_Q8S1T0      SPLKRHVSRTVDFVHENWKFIWLVALWLAVNVGLFAYKFEQYERRAAFQVMGHCVCVAKG 332
OsRbohF_Q0J595      WSPRRAAARAQVAAEENWRFAWVLALWFAAMAGLFAWKFVQYRRTPAFRVMGYCLPTAKG 454
OsRbohG_Q69LJ7      WGVRKAAARVRVAAEENWRFAWVVALWFAAMASLFVWKFVQYRRTPAFRVMGYCLPTAKG 472
OsRbohH_Q2QP56      NPLRRGLRRLSYFMEDNWKFVWVMALWLAINAGLFTWKFMAYKRHPTFDVMGYCVCVAKG 394
OsRbohI_Q2R351      NPLRRWWRRASYFLEDNWRFVWVLLLWLAICAGLFTYKFIQYRHRAVFHVMGYCVCVAKG 423
AtRbohA_O81209      NLGARFCRGMKYFLFDNWKFVWVMALWIGAMAGLFTWKFMEYRKRSAYEVMGVCVCIAKG 384
AtRbohB_Q9SBI0      NPVKRFAMNISYFFLENWKFIWVLTLWISICITLFTWKFLQYKRKTVFEVMGYCVTVAKG 337
AtRbohC_O81210      NPLKRWYRGLRFFLLDNWQFCWVIVLWFIVMAILFTYKYIQYRRSPVYPVMGDCVCMAKG 391
AtRbohD_Q9FIJ0      NPLLRWSEKIKYFILDNWQFLWIMMLWLGICGGLFTYKFIQYKNKAAYGVMGYCVCVAKG 416
AtRbohE_O81211      HVVQKCRKKLQCLILDNWQFSWVLLVWVMLMAILFVWKFLEYREKAAFKVMGYCLTTAKG 432
AtRbohF_O48538      SRIHRMSSDFVYIMQENWKFIWVLSLWIMIMIGLFLWKFFQYKQKDAFHVMGYCLLTAKG 427
AtRbohG_Q9SW17      NHLRRWYCQLRFFVLDSWQFVWVIALWLTIMAILFAYKYIQYKNRAVYEVLGPCVCLAKG 343
AtRbohH_Q9FJD6      TPMSKYVSVTAELMHENWKFLWVLALWAIINVYLFMWKYEEFMRNPLYNITGRCVCAAKG 356
AtRbohI_Q9SUT8      ---WRMSRNLLYSLQDNWKFIWVLTLWFVIMAWLFMWKCYQYKHKDAFHVMGYCLVMAKG 414
AtRbohJ_Q9LZU9      TPTSKYVVVTAELMYEHWKFIWVVTLWLAVNVVLFMWKYEEFTTSPLYNITGRCLCAAKG 367

                        TMD-II            B-loop           TMD-III
OsRbohA_Q0JJJ9      AAETLKLNMAIILLPVCRNTITWLRSTR--AARALPFDDNINFHKT-------------- 265
OsRbohB_Q5ZAJ0      AAETLKFNMALVLLPVCRNTITWIRSKTQ-VGAVVPFNDNINFHKV-------------- 440
OsRbohC_Q65XC8      AAETLKLNMAIILLPVCRNTITWLRNTR--AARALPFDDNINFHKT-------------- 473
OsRbohD_Q0DHH6      AAETLKLNMAVILLPVCRNTLTRLRSTA--LSKVVPFDDNINFHKV-------------- 351
OsRbohE_Q8S1T0      AAEVLKLNMALILLPVCRNTLTTLRSTA--LSHVIPFDDNINFHKV-------------- 376
OsRbohF_Q0J595      AAETLKLNMALVLLPVCRNTLTWLRSSW--ARFFVPFDDSITFHKVESSSGFKWAPPGRS 512
OsRbohG_Q69LJ7      AAETLKLNMALVLLPVCRNTLTWLRSSW--ARFFVPFDDNITFHKM-------------- 516
OsRbohH_Q2QP56      GAETTKFNMALILLPVCRNTITWLRSRTK-LGAVIPFNDNINFHKV-------------- 439
OsRbohI_Q2R351      GAETLKFNMALILLPVCRNTVTWIRNRAA-VARVVPFDDNINFHKV-------------- 468
AtRbohA_O81209      AAETLKLNMAMILLPVCRNTITWLRTKTK-LSAIVPFDDSINFHKV-------------- 429
AtRbohB_Q9SBI0      SAETLKFNMALILLPVCRNTITWLRTKSKLIGSVVPFDDNINFHKV-------------- 383
AtRbohC_O81210      AAETVKLNMALILLPVCRNTITWLRNKTR-LGRVVPFDDNINFHKV-------------- 436
AtRbohD_Q9FIJ0      GAETLKFNMALILLPVCRNTITWLRNKTK-LGTVVPFDDSINFHKV-------------- 461
AtRbohE_O81211      AAETLKLNMALVLLPVCRNTLTWLRSTR--ARACVPFDDNINFHKI-------------- 476
AtRbohF_O48538      AAETLKFNMALILFPVCRNTITWLRSTR--LSYFVPFDDNINFHKT-------------- 471
AtRbohG_Q9SW17      AAETLKLNMALILLPVCRNTITWLRNKTR-LGVFVPFDDNINFHKV-------------- 388
AtRbohH_Q9FJD6      AAETLKLNMALILVPVCRKTLTILRSTF--LNRVVPFDDNINFHKV-------------- 400
AtRbohI_Q9SUT8      AAETLKFNMALILLPVCRNTITYLRSTA--LSHSVPFDDCINFHKT-------------- 458
AtRbohJ_Q9LZU9      TAEILKLNMALILVPVLRRTLTFLRSTF--LNHLIPFDDNINFHKL-------------- 411

OsRbohA_Q0JJJ9      ------------------------------------------IAAAIVVGIILHAGNH 281
OsRbohB_Q5ZAJ0      ------------------------------------------IAAGVAVGVALHAGAH 456
OsRbohC_Q65XC8      ------------------------------------------IAAAIVVGVILHGGLH 489
OsRbohD_Q0DHH6      ------------------------------------------IALTIAIGAATHTLAH 367
OsRbohE_Q8S1T0      ------------------------------------------IAATIAAATAVHTLAH 392
OsRbohF_Q0J595      NQCSVAGEPAGTEDPRPGRLRVKAPVIQKSPRPTGLPLSCGATIIATAIALGICTHAGTH 572
OsRbohG_Q69LJ7      ------------------------------------------IATAIVVGITLHAGNH 532
OsRbohH_Q2QP56      ------------------------------------------VAGGVVVGVALHGVTH 455
OsRbohI_Q2R351      ------------------------------------------IAVGITVGAGLHVISH 484
AtRbohA_O81209      ------------------------------------------IAIGISVGVGIHATSH 445
AtRbohB_Q9SBI0      ------------------------------------------VAFGIAVGIGLHAISH 399
AtRbohC_O81210      ------------------------------------------IAVGIIVGVTMHAGAH 452
AtRbohD_Q9FIJ0      ------------------------------------------IASGIVVGVLLHAGAH 477
AtRbohE_O81211      ------------------------------------------IACAIAIGILVHAGTH 492
AtRbohF_O48538      ------------------------------------------IAGAIVVAVILHIGDH 487
AtRbohG_Q9SW17      ------------------------------------------IAVGIAIGVAIHSVSH 404
AtRbohH_Q9FJD6      ------------------------------------------IAYMIAFQALLHTALH 416
AtRbohI_Q9SUT8      ------------------------------------------ISVAIISAMLLHATSH 474
AtRbohJ_Q9LZU9      ------------------------------------------IAVAIAVISLLHTALH 427
```

```
                                            C-loop                          TMD-IV
OsRbohA_Q0JJJ9    LVCDFPRLIKSSDEKYAP-LGQYFGEIK-PTYFTLVKGVEGITGVIMVVCMIIAFTLATR 339
OsRbohB_Q5ZAJ0    LTCDFPRLLHASDAQYEL-MKPFFGEKRPPNYWWFVKGTEGWTGVVMVVLMAIAFTLAQP 515
OsRbohC_Q65XC8    LVCDFPRLIGSSEEKYAP-LGKYFGETK-PTYLTLVKGVEGITGVIMLVCMIIAFTLATR 547
OsRbohD_Q0DHH6    VTCDFPRLVSCPRDKFEATLGPYFNYVQ-PTYSSLVASTHGWTGILMILIMSFSFTLATH 426
OsRbohE_Q8S1T0    VTCDFPRLINCPSDKFMATLGPNFGYRQ-PTYADLLESAHGVTGILMIIIMSFSFTLATH 451
OsRbohF_Q0J595    LACDFPRLIGSSREEYELLLSGFFGASR-PTYRGLLAGVEGVTGIVMVVLMVVSFTLATR 631
OsRbohG_Q69LJ7    LACDFPRLIASGPEEYRLVA-DAFGPEK-PTYVGLLSGVEGITGVAMVVLMTVSFTLATH 590
OsRbohH_Q2QP56    LTCDFPRLLHASDAAYEP-MKKYFGQTRIPDYWWFVRGVEGITGVIMVVLMAIAYTLAHP 514
OsRbohI_Q2R351    LTCDFPRLLHATDAEYEP-MKRFFGDTRPPNYWWFVKGTEGWTGLVMLVLMAVAFTLATP 543
AtRbohA_O81209    LACDFPRLIAADEDQYEP-MEKYFGP-QTKRYLDFVQSVEGVTGIGMVVLMTIAFTLATT 503
AtRbohB_Q9SBI0    LACDFPRLLHAKNVEFEP-MKKFFGDERPENYGWFMKGTDGWTGVTMVVLMLVAYVLAQS 458
AtRbohC_O81210    LACDFPRLLHATPEAYRP-LRQFFGDEQPKSYWHFVNSVEGITGLVMVLLMAIAFTLATP 511
AtRbohD_Q9FIJ0    LTCDFPRLIAADEDTYEP-MEKYFGD-QPTSYWWFVKGVEGWTGIVMVVLMAIAFTLATP 535
AtRbohE_O81211    LACDFPRIINSSPEQFVL-IASAFNGTK-PTFKDLMTGAEGITGISMVILTTIAFTLAST 550
AtRbohF_O48538    LACDFPRIVRATEYDYNRYLFHYFQTKQ-PTYFDLVKGPEGITGILMVILMIISFTLATR 546
AtRbohG_Q9SW17    LACDFPLLIAATPAEYMP-LGKFFGEEQPKRYLHFVKSTEGITGLVMVFLMVIAFTLAMP 463
AtRbohH_Q9FJD6    IFCNYPRLSSCSYDVFLTYAGAALGNTQ-PSYLGLMLTSVSITGVLMIFFMGFSFTLAMH 475
AtRbohI_Q9SUT8    LACDFPRILASTDTDYKRYLVKYFGVTR-PTYFGLVNTPVGITGIIMVAFMLIAFTLASR 533
AtRbohJ_Q9LZU9    MLCNYPRLSSCPYNFYSDYAGNLLGAKQ-PTYLGLMLTPVSVTGVLMILFMGISFTLAMH 486

                             D-loop                               TMD-V
OsRbohA_Q0JJJ9    WFRRS----------------LVK--LPRPFDKLTGFNAFWYSHHLFIIVYIALIVHGEC 381
OsRbohB_Q5ZAJ0    WFRRN----------------KLK--DSNPLKKMTGFNAFWFTHHLFVIVYTLLFVHGTC 557
OsRbohC_Q65XC8    WFRRS----------------LVK--LPKPFDKLTGFNAFWYSHHLFIIVYISLVIHGEW 589
OsRbohD_Q0DHH6    SFRRS----------------VVK--LPSPLHHLAGFNAFWYAHHLLVIAYILLVLHSYF 468
OsRbohE_Q8S1T0    SFRRS----------------VVK--LPSPLHHLAGFNAFWYAHHLLVLAYVLLVVHSYF 493
OsRbohF_Q0J595    PLRKRE---------------APR--LPFPLGHLAGFNAFWYSHHLLIVVYLLLLVHGWF 674
OsRbohG_Q69LJ7    PFRKGEKGGSGGGAAATVLPTVAR--LPSPFNRLAGFNAFWYSHHLLGIVYALLIAHGYF 648
OsRbohH_Q2QP56    WFRRS----------------KLS--DSNPLKRLSGFNMFWYSHHLFVIVYIAFVVHGVC 556
OsRbohI_Q2R351    WFRRG----------------RLR--LPRPLNRLTGFNAFWYSHHCFVIVYALLIVHGYY 585
AtRbohA_O81209    WFRRN----------------KLN--LPGPLKKITGFNAFWYSHHLFVIVYSLLVVHGFY 545
AtRbohB_Q9SBI0    WFRRN----------------RAN--LPKSLKRLTGFNAFWYSHHLFVIVYVLLIVHGYF 500
AtRbohC_O81210    WFRRG----------------KLN-YLPGPLKKLASFNAFWYTHHLFVIVYILLVAHGYY 554
AtRbohD_Q9FIJ0    WFRRN----------------KLN--LPNFLKKLTGFNAFWYTHHLFIIVYALLIVHGIK 577
AtRbohE_O81211    HFRRN----------------RVR--LPAPLDRLTGFNAFWYTHHLLVVVYIMLIVHGTF 592
AtRbohF_O48538    WFRRN----------------LVK--LPKPFDRLTGFNAFWYSHHLFVIVYILLILHGIF 588
AtRbohG_Q9SW17    WFRRG----------------KLEKKLPGPLKKLASFNAFWYTHHLFVIVYILLVLHGYY 507
AtRbohH_Q9FJD6    YFRRN----------------IVK--LPKPFNVLAGFNAFWYAHHLLVLAYILLIIHGYY 517
AtRbohI_Q9SUT8    RCRRN----------------LTK--LPKPFDKLTGYNAFWYSHHLLLTVYVLLVIHGVS 575
AtRbohJ_Q9LZU9    YFRRN----------------IVK--LPIPFNRLAGFNSFWYAHHLLVIAYALLIIHGYI 528

                     E-loop      TMD-VI
OsRbohA_Q0JJJ9    LYLI-HVWYRRTTWMYLSVPVCLYVGERILRFF-RSG-SYSVRLLKVAIYPGNVLTLQMS 438
OsRbohB_Q5ZAJ0    LYLS-RKWYKKTTWMYLAVPVVLYVSERILRLF-RSH--DAVGIQKVAVYPGNVLALYMS 613
OsRbohC_Q65XC8    LYLI-RIWYKRTTWMYLAVPVCLYVGERTLRFF-RSG-SYSVRLLKVAIYPGNVLTLQMS 646
OsRbohD_Q0DHH6    IFLT-KQWYNRTTWMFLAVPVLFYSCERTIRRV-RES-SYGVTVIKAAIYPGNVLSIHMN 525
OsRbohE_Q8S1T0    IFLT-REWYKKTTWMYLIVPVLFYACERTIRKV-REN-NYRVSIVKAAIYPGNVLSLHMK 550
OsRbohF_Q0J595    MFLV-TKWHQRTTWMYIAVPLMLYVGERTLRAF-RSK-AYAVKILKVCLLPGNVLTITMS 731
OsRbohG_Q69LJ7    LFLV-RRWYLKTTWMYISVPLMLYVGERMLRAL-RSN-AYAVKILKVCLLPGNVLTITMS 705
OsRbohH_Q2QP56    LYIN-RTWWKQTTWMYLAIPILLYAGERIFRAL-RSHGFTTVRIEKVAIYPGNVIAIHMT 614
OsRbohI_Q2R351    LFLT-KDWYKKTTWMYLAVPMFLYACERLTRAL-RSS-VRPVKILKVAVYPGNVLSLHFS 642
AtRbohA_O81209    VYLIIEPWYKKTTWMYLMVPVVLYLCERLIRAF-RSS-VEAVSVLKVAVLPGNVLSLHLS 603
AtRbohB_Q9SBI0    VYLS-KEWYHKTTWMYLAVPVLLYAFERLIRAF-RPG-AKAVKVLKVAVYPGNVLSLYMS 557
AtRbohC_O81210    LYLT-RDWHNKTTWMYLVVPVVLYACERLIRAF-RSS-IKAVTIRKVAVYPGNVLAIHLS 611
AtRbohD_Q9FIJ0    LYLT-KIWYQKTTWMYLAVPILLYASERLLRAF-RSS-IKPVKMIKVAVYPGNVLSLHMT 634
AtRbohE_O81211    LFFA-DKWYQKTTWMYISVPLVLYVAERSLRAC-RSK-HYSVKILKVSMLPGEVLSLIMS 649
AtRbohF_O48538    LYFA-KPWYVRTTWMYLAVPVLLYGGERTLRYF-RSG-SYSVRLLKVAIYPGNVLTLQMS 645
AtRbohG_Q9SW17    IYLN-KEWYKKTTWMYLAVPVALYAYERLIRAF-RSS-IRTVKVLKMAAYPGKVLTLQMS 564
AtRbohH_Q9FJD6    LIIE-KPWYQKTTWMYLAVPMLFYASERLFSRLLQEH-SHRVNVIKAIVYSGNVLALYVT 575
AtRbohI_Q9SUT8    LYLE-HKWYRKTVWMYLAVPVLLYVGERIFRFF-RSR-LYTVEICKVVIYPGNVVVLRMS 632
AtRbohJ_Q9LZU9    LIIE-KPWYQKTTWMYVAIPMVLYASERLFSRV-QEH-NHRVHIIKAIVYSGNVLALYMT 585
```

```
                                                FAD-I            FAD-II

OsRbohA_Q0JJJ9   KPPTFRYKSGQYMFVQCPAVSPFEWHPFSITSAPGDDYLSIHVRQLGDWTRELKRVFAAA 498
OsRbohB_Q5ZAJ0   KPPGFRYRSGQYIFIKCTAVSPYEWHPFSITSAPGDDYLSVHIRTRGDWTSRLRTVFSEA 673
OsRbohC_Q65XC8   KPPTFRYKSGQYMFVQCPAVSPFEWHPFSITSAPGDDYLSIHVRQLGDWTRELKRVFSAA 706
OsRbohD_Q0DHH6   KPSSFKYKSGMYMFVKCPDVSPFEWHPFSITSAPGDDYLSVHIRTLGDWTTELRNLFGKA 585
OsRbohE_Q8S1T0   KPPGFKYKSGMYLFVKCPDVSPFEWHPFSITSAPGDDYLSVHIRTLGDWTTELRNLFGKA 610
OsRbohF_Q0J595   KPYGFRYRSGQYIFLQCPTISPFEWHPFSITSAPGDDYISVHIQTRGDWTQELKRIFVEN 791
OsRbohG_Q69LJ7   KPYGFRYRSGQYIFLQCPTISPFEWHPFSITSAPGDDYLSVHIRTNGDWTQELKRIFVEN 765
OsRbohH_Q2QP56   KPHGFKYKSGQYIYVNCGEVSPFEWHPFTITSAPDDSYLSMHIRCRGDWTSSFRAIFSQI 674
OsRbohI_Q2R351   KPQGFKYKSGQYIFVNCAAVSPFQWHPFSITSAPQDDYVSVHIRTLGDWTRELKNVFSRV 702
AtRbohA_O81209   RPSNFRYKSGQYMYLNCSAVSTLEWHPFSITSAPGDDYLSVHIRVLGDWTKQLRSLFSEV 663
AtRbohB_Q9SBI0   KPKGFKYTSGQYIYINCSDVSPLQWHPFSITSASGDDYLSVHIRTLGDWTSQLKSLYSKV 617
AtRbohC_O81210   RPQNFKYKSGQYMFVNCAAVSPFEWHPFSITSAPQDDYLSVHIRVLGDWTRALKGVFSEV 671
AtRbohD_Q9FIJ0   KPQGFKYKSGQFMLVNCRAVSPFEWHPFSITSAPGDDYLSVHIRTLGDWTRKLRTVFSEV 694
AtRbohE_O81211   KPPGFKYKSGQYIFLQCPTISRFEWHPFSITSAPGDDQLSVHIRTLGDWTEELRRVLTVG 709
AtRbohF_O48538   KPTQFRYKSGQYMFVQCPAVSPFEWHPFSITSAPEDDYISIHIRQLGDWTQELKRVFSEV 705
AtRbohG_Q9SW17   KPTNFKYMSGQYMFVNCPAVSPFEWHPFSITSTPQDDYLSVHIKALGDWTEAIQGVFSEV 624
AtRbohH_Q9FJD6   KPPGFKYKSGMYMFVKCPDLSKFEWHPFSITSAPGDDYLSVHIRALGDWTTELRSRFAKT 635
AtRbohI_Q9SUT8   KPTSFDYKSGQYVFVQCPSVSKFEWHPFSITSSPGDDYLSIHIRQRGDWTEGIKKAFSVV 692
AtRbohJ_Q9LZU9   KPQGFKYKSGMYMFVKCPDISKFEWHPFSITSAPGDEYLSVHIRALGDWTSELRNRFAET 645


OsRbohA_Q0JJJ9   CEP--PAGG------------KSGLLRA-----DETTKKILPKLLIDGPYGSPAQDYSKY 539
OsRbohB_Q5ZAJ0   CRPP--TEG------------ESGLLRADLSKGITDEKARFPKLLVDGPYGAPAQDYREY 719
OsRbohC_Q65XC8   CEP--PVGG------------KSGLLRA-----DETTKKALPKLLIDGPYGSPAQDYSKY 747
OsRbohD_Q0DHH6   CEAQVSSKKA---TLARLETTII---AD-----GLKEETCFPKVFIDGPFGAPAQNYKKY 634
OsRbohE_Q8S1T0   CEAQVTSKKA---TLSRLETTVV---AD-----AQTEDTRFPKVLIDGPYGAPAQNYKKY 659
OsRbohF_Q0J595   YFV-PSVPR------------RASFGAL-----GMAEQKSPPRLLVDGPYGAPAQDFRNY 833
OsRbohG_Q69LJ7   YFS-PHLNR------------RASFSEL-----GATEPRSLPRLLVDGPYGAPAQDFRNY 807
OsRbohH_Q2QP56   CRPP--MNG------------QSGLLRADCMS--MEHHSRFPKLLIDGPYGAPAQDYWKY 718
OsRbohI_Q2R351   CRPP--TEG------------KSGLLRAEYDRDGAMTNPSFPKVLIDGPYGAPAQDYKQY 748
AtRbohA_O81209   CKPR--PPD------------EHRLNRADSKH--WDYIPDFPRILIDGPYGAPAQDYKKF 707
AtRbohB_Q9SBI0   CQLP--STS------------QSGLFIADIGQA--NNITRFPRLLIDGPYGAPAQDYRNY 661
AtRbohC_O81210   CKPP--PAG------------VSGLLRADMLH--GANNPDFPKVLIDGPYGAPAQDYKKY 715
AtRbohD_Q9FIJ0   CKPP--TAG------------KSGLLRAD----GGDGNLPFPKVLIDGPYGAPAQDYKKY 736
AtRbohE_O81211   KDLSTCVIG------------RSKFSAY-----CNIDMINRPKLLVDGPYGAPAQDYRSY 752
AtRbohF_O48538   CEP--PVGG------------KSGLLRA-----DETTKKSLPKLLIDGPYGAPAQDYRKY 746
AtRbohG_Q9SW17   SKPP--PVG-------------------DMLN--GANSPRFPKIMIDGPYGAPAQDYKKY 661
AtRbohH_Q9FJD6   CEPTQAAAKPKPNSLMRMETRAAGVNPH-----IEESQVLFPKIFIKGPYGAPAQNYQKF 690
AtRbohI_Q9SUT8   CHA--PEAG------------KSGLLRA-----DVPNQRSFPELLIDGPYGAPAQDHWKY 733
AtRbohJ_Q9LZU9   CEPHQK-SKPSPNDLIRMETRARGANPH-----VEESQALFPRIFIKGPYGAPAQSYQKF 699


                   NADPH-I
OsRbohA_Q0JJJ9   DVLLLVGLGIGATPFISILKDLLNNIIKMEEEEDAST-DLYPPMGRN--KP----HVDLG 592
OsRbohB_Q5ZAJ0   DVLLLIGLGIGATPLISIVKDVLNHIQGEGSVGTT---------E--------------- 755
OsRbohC_Q65XC8   DVLLLVGLGIGATPFISILKDLINSIIKMEEEEEASG-DLYPPIGRN--KA----HVDLD 800
OsRbohD_Q0DHH6   DILLLIGLGIGATPFISILKDLLNNIKSNGDVQ------STHDAELGC------------ 676
OsRbohE_Q8S1T0   DILLLIGLGIGATPFISILKDLLNNIKSNEEVE------SIHGSEIG------------- 700
OsRbohF_Q0J595   DVLLLVGLGIGATPFISILRDLLNNIKLADELMDLAMET-S----RS------DDSANSF 882
OsRbohG_Q69LJ7   DVLLLVGLGIGATPFISILRDLLNNIKLAEELMDLAMET-S----RS------EDSANSF 856
OsRbohH_Q2QP56   DVLLLIGLGIGATPLISIVKDVLNHIYDDPESAASPHT--TN------------------ 758
OsRbohI_Q2R351   DIVLLVGLGIGATPMISIIKDIINNMRQLDGDLED-------GDG--------------- 786
AtRbohA_O81209   EVVLLVGLGIGATPMISIVSDIINNLKGVEEGSNRRQSPIHNMVT--------------- 752
AtRbohB_Q9SBI0   DVLLLIGLGIGATPLISIIRDVLNNIKNQNSIE----------RG--------------- 696
AtRbohC_O81210   EVVLLVGLGIGATPMISIVKDIVNNIKAKEQAQLNRME-----NG--------------- 755
AtRbohD_Q9FIJ0   DVVLLVGLGIGATPMISILKDIINNMKGPDRDSDI------------------------- 771
AtRbohE_O81211   DVLLLIGLGIGATPFISILKDLLNNSRDEQTDNEFSRSDFSWN-----------SCTSSY 801
AtRbohF_O48538   DVLLLVGLGIGATPFISILKDLLNNIVKMEEHADSIS-DFSRSSEYS--T---------- 793
AtRbohG_Q9SW17   EVVLLIGLGIGATPMISIIKDIINNTETKE--QLSQME-----KG--------------- 699
AtRbohH_Q9FJD6   DILLLVGLGIGATPFISILKDMLNHLKPGIPRSGQKYEGSVGGESIGGDSVSG------- 743
AtRbohI_Q9SUT8   DVVLLVGLGIGATPFVSILRDLLNNIIKQQEQAECIS-GSCSNSNIS--SDHSFSCLNSE 790
AtRbohJ_Q9LZU9   DILLLIGLGIGATPFISILKDMLNNLKPGIPKTGQKYEGSVGGESLGGSSVYGGSSVNGG 759
```

```
                                      NADPH-II
OsRbohA_Q0JJJ9    T--LMTITSRPKKILKTTNAYFYWVTREQGSFDWFKGVMNEIADLDQR-NIIEMHNYLTS 649
OsRbohB_Q5ZAJ0    ---PESSSKAKKKPFMTKRAYFYWVTREEGSFEWFRGVMNEVSEKDKD-GVIELHNHCSS 811
OsRbohC_Q65XC8    T--LMRITSKPKRVLKTTNAYFYWVTREQGSFDWFKGVMNEIAELDQR-NIIEMHNYLTS 857
OsRbohD_Q0DHH6    ----------TFKSNGPGRAYFYWVTREQGSFEWFKGVMNDVAESDHD-NVIEMHNYLTS 725
OsRbohE_Q8S1T0    ----------SFKNNGPGRAYFYWVTREQGSFEWFKGVMNDVAESDHN-NIIEMHNYLTS 749
OsRbohF_Q0J595    S--VSTASSNKRRAYRTSRAHFYWVTREPGSFEWFKGVMNEVAEMDKK-GVIELHNYLTS 939
OsRbohG_Q69LJ7    S--VSTASSNKKRAYRTSRAHFYWVTREPLSFEWFKGVMNEVAEMDKK-GVIELHNYLTS 913
OsRbohH_Q2QP56    ---GGGAAAAARRAFMTKRVYFYWCTREEGSFEWFRGVMNEVADRDAGRELIELHNHCTS 815
OsRbohI_Q2R351    ---NDNSVSSSSAAFKTRRAYFYWVTREQGSFEWFRGVMDEVAETDKK-GVIELHNYCTS 842
AtRbohA_O81209    ---PPVSPSRKSETFRTKRAYFYWVTREQGSFDWFKNVMDEVTETDRK-NVIELHNYCTS 808
AtRbohB_Q9SBI0    ---TNQ---HIKNYVATKRAYFYWVTREQGSLEWFSEVMNEVAEYDSE-GMIELHNYCTS 749
AtRbohC_O81210    ---TSEPQRSKKESFRTRRAYFYWVTREQGSFDWFKNIMNEVAERDAN-RVIEMHNYCTS 811
AtRbohD_Q9FIJ0    ---ENNNSNNNSKGFKTRKAYFYWVTREQGSFEWFKGIMDEISELDEE-GIIELHNYCTS 827
AtRbohE_O81211    T--TATPTSTHGGKKKAVKAHFYWVTREPGSVEWFRGVMEEISDMDCR-GQIELHNYLTS 858
AtRbohF_O48538    G--SNGDTPRRKRILKTTNAYFYWVTREQGSFDWFKGVMNEVAELDQR-GVIEMHNYLTS 850
AtRbohG_Q9SW17    ---SPQEQQGNKETFKTRRAYFYWVTKEQGTFDWFKNIMNEIAERDKS-KVIELHNHCTS 755
AtRbohH_Q9FJD6    ----------GGGKKFPQRAYFFWVTREQASFDWFKGVMDDIAEYDKT-HVIEMHNYLTS 792
AtRbohI_Q9SUT8    A--ASRIPQTQRKTLNTKNAYFYWVTREQGSFDWFKEIMNEIADSDRK-GVIEMHNYLTS 847
AtRbohJ_Q9LZU9    GSVNGGGSVSGGGRKFPQRAYFYWVTREQASFEWFKGVMDDIAVYDKT-NVIEMHNYLTS 818

                                             NADPH-III
OsRbohA_Q0JJJ9    VYEEGDARSALITMLQALNHAKNGVDIVSGTKVRTHFARPNWRKVLSKISSKHPYAKIGV 709
OsRbohB_Q5ZAJ0    VYQEGDARSALIVMLQELQHAKKGVDILSGTSVKTHFARPNWRSVFKKVAVSHENQRVGV 871
OsRbohC_Q65XC8    VYEEGDARSALITMLQALNHAKNGVDIVSGTKVRTHFARPNFKKVLSKIASKHPYAKIGV 917
OsRbohD_Q0DHH6    VYEEGDARSALIAMVQSLQHAKNGVDIVSGSKIRTHFARPNWRKVFSDLANAHQNSRIGV 785
OsRbohE_Q8S1T0    VYEEGDARSALIAMVQSLQHAKNGVDIVSGSRIRTHFARPNWRKVFSDLANAHKNSRIGV 809
OsRbohF_Q0J595    VYEERDARSTLLSMVQALNHAKHGVDIVSGTRVRTHFARPNWKEVFTRIASKHPNSTVGV 999
OsRbohG_Q69LJ7    VYEERDARTTLLSMVQALNHAKHGVDIVSGTRVRTHFARPNWKEVFTRIASKHPNSTVGV 973
OsRbohH_Q2QP56    VYEEGDARSALVTMLQALHHAKNGVDVVSGTRVRTHFARPSWRDVFKRVAVNHQGQRVGV 875
OsRbohI_Q2R351    VYEEGDARSALIAMLQSLNHAKHGVDVVSGTRVKTHFARPNWRNVYKRIALNHRDQRVGV 902
AtRbohA_O81209    VYEEGDARSALITMLQSLNHAKHGVDVVSGTRVMSHFARPNWRSVFKRIAVNHPKTRVGV 868
AtRbohB_Q9SBI0    VYEEGDARSALITMLQSLHHAKSGIDIVSGTRVRTHFARPNWRSVFKHVAVNHVNQRVGV 809
AtRbohC_O81210    VYEEGDARSALIHMLQSLNHAKNGVDIVSGTRVMSHFAKPNWRNVYKRIAMDHPNTKVGV 871
AtRbohD_Q9FIJ0    VYEEGDARVALIAMLQSLQHAKNGVDVVSGTRVKSHFAKPNWRQVYKKIAVQHPGKRIGV 887
AtRbohE_O81211    VYDEGDARSTLIKMVQALNHAKHGVDILSGTRVRTHFARPNWKEVFSSIARKHPNSTVGV 918
AtRbohF_O48538    VYEEGDARSALITMVQALNHAKNGVDIVSGTRVRTHFARPNWKKVLTKLSSKHCNARIGV 910
AtRbohG_Q9SW17    VYEEGDVRSALIRMLQSLNYAKNGLDIVAGTRVMSHFARPNWKNVYKQIAMDHPGANVGV 815
AtRbohH_Q9FJD6    MYEAGDARSALIAMVQKLQHAKNGVDIVSESRIRTHFARPNWRKVFSELSSKHEACRIGV 852
AtRbohI_Q9SUT8    VYEEGDTRSNLLTMIQTLNHAKNGVDIFSGTKVRTHFGRPKWKKVLSKISTKHRNARIGV 907
AtRbohJ_Q9LZU9    MYEAGDARSALIAMVQKLQHAKNGVDIVSESRIRTHFARPNWRKVFSELSNKHETSRIGV 878

                 NADPH-IV
OsRbohA_Q0JJJ9    FYCGAPVLAQELSKLCHEFNGKCTTKFEFHKEHF 743
OsRbohB_Q5ZAJ0    FYCGEPVLVPQLRQLSADFTHKTNTRFDFHKENF 905
OsRbohC_Q65XC8    FYCGAPVLAQELSDLCHDFNGRCTSKFEFHKEHF 951
OsRbohD_Q0DHH6    FYCGSPTLTKTMLRDLSLEFSQTTTTRFHFHKENF 819
OsRbohE_Q8S1T0    FYCGSPTLTKQLKDLSKEFSQTTTTRFHFHKENF 843
OsRbohF_Q0J595    FYCGKPTLAKELKKLSLDMSHKTTTRFHFHKEYF 1033
OsRbohG_Q69LJ7    FYCGAPTLAKELKTLSHEMSHRTGTRFHFHKEYF 1007
OsRbohH_Q2QP56    FFCGDQALTPELRRLAQDFSHKTTTKFVFHKENF 909
OsRbohI_Q2R351    FYCGAPVLTKELRELAQDFSRKTSTKFDFHKENF 936
AtRbohA_O81209    FYCGAAGLVKELRHLSLDFSHKTSTKFIFHKENF 902
AtRbohB_Q9SBI0    FYCGNTCIIGELKRLAQDFSRKTTTKFEFHKENF 843
AtRbohC_O81210    FYCGAPALTKELRHLALDFTHKTSTRFSFHKENF 905
AtRbohD_Q9FIJ0    FYCGMPGMIKELKNLALDFSRKTTTKFDFHKENF 921
AtRbohE_O81211    FYCGIQTVAKELKKQAQDMSQKTTTRFEFHKEHF 952
AtRbohF_O48538    FYCGVPVLGKELSKLCNTFNQKGSTKFEFHKEHF 944
AtRbohG_Q9SW17    FYCGAPVLTKELRQLALEFTHKTSTRFSFHKENF 849
AtRbohH_Q9FJD6    FYCGSPTLVRPLKELCQEFSLESSTRFTFHKENF 886
AtRbohI_Q9SUT8    FYCGVPSLGKELSTLCHEFNQTGITRFDFHKEQF 941
AtRbohJ_Q9LZU9    FYCGSPTLVRPLKSLCQEFSLESSTRFTFHKENF 912
```
